# 4-[(1,3-Dioxoisoindolin-2-yl)meth­yl]benzene­sulfonamide

**DOI:** 10.1107/S1600536814002803

**Published:** 2014-02-12

**Authors:** Joel T. Mague, Alaa A.-M. Abdel-Aziz, Adel S. El-Azab, Magda A. El-Sherbeny

**Affiliations:** a Department of Chemistry, Tulane University, New Orleans, LA 70118, USA; b Department of Pharmaceutical Chemistry, College of Pharmacy, King Saud University, Riyadh 11451, Saudi Arabia; c Department of Medicinal Chemistry, Faculty of Pharmacy, University of Mansoura, Mansoura 35516, Egypt; d Department of Organic Chemistry, Faculty of Pharmacy, Al-Azhar University, Cairo 11884, Egypt

## Abstract

The title compound, C_15_H_12_N_2_O_4_S, is V-shaped with the isoindoline ring system (r.m.s. deviation = 0.006 Å) inclined to the benzene ring by 84.27 (13)°. In the crystal, inversion dimers are formed *via* pairwise N—H⋯O hydrogen bonds. These dimers associate further into corrugated ribbons, *via* pairwise N—H⋯O and C—H⋯O hydrogen bonds, propagating along the *a*-axis direction and lying parallel to (001).

## Related literature   

For the biological activity of cyclic imides, see: Abdel-Aziz *et al.* (2011*a* ▶,*b* ▶); Abdel-Aziz (2007 ▶). For related crystal structures, see: Jiang *et al.* (2008 ▶); Li (2007 ▶); Warzecha *et al.* (2006 ▶). For the preparation of the title compound, see: Abdel-Aziz *et al.* (2011*a* ▶).
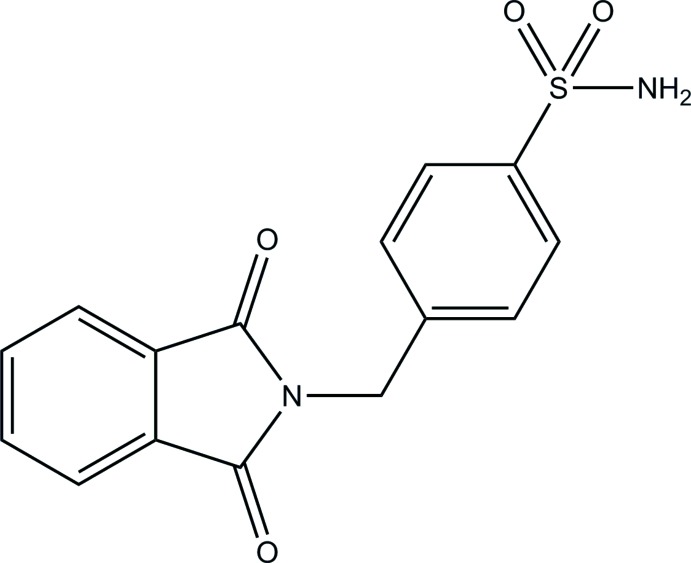


## Experimental   

### 

#### Crystal data  

C_15_H_12_N_2_O_4_S
*M* *_r_* = 316.33
Monoclinic, 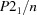

*a* = 4.9803 (1) Å
*b* = 26.5291 (7) Å
*c* = 10.2740 (3) Å
β = 90.804 (1)°
*V* = 1357.30 (6) Å^3^
*Z* = 4
Cu *K*α radiation
μ = 2.33 mm^−1^
*T* = 100 K
0.27 × 0.07 × 0.02 mm

#### Data collection  

Bruker D8 VENTURE PHOTON 100 CMOS diffractometer
Absorption correction: multi-scan (*SADABS*; Bruker, 2012 ▶) *T*_min_ = 0.85, *T*_max_ = 0.95
22413 measured reflections
2533 independent reflections
2277 reflections with *I* > 2σ(*I*)
*R*_int_ = 0.036

#### Refinement  

*R*[*F* ^2^ > 2σ(*F* ^2^)] = 0.042
*wR*(*F* ^2^) = 0.097
*S* = 1.17
2533 reflections
207 parameters
60 restraints
H atoms treated by a mixture of independent and constrained refinement
Δρ_max_ = 0.35 e Å^−3^
Δρ_min_ = −0.48 e Å^−3^

### 

Data collection: *APEX2* (Bruker, 2012 ▶); cell refinement: *SAINT* (Bruker, 2012 ▶); data reduction: *SAINT*; program(s) used to solve structure: *SHELXS97* (Sheldrick, 2008 ▶); program(s) used to refine structure: *SHELXL2013* (Sheldrick, 2008 ▶); molecular graphics: *DIAMOND* (Brandenburg & Putz, 2012 ▶); software used to prepare material for publication: *SHELXTL* (Sheldrick, 2008 ▶).

## Supplementary Material

Crystal structure: contains datablock(s) I, global. DOI: 10.1107/S1600536814002803/su2697sup1.cif

Structure factors: contains datablock(s) I. DOI: 10.1107/S1600536814002803/su2697Isup2.hkl

Supporting information file. DOI: 10.1107/S1600536814002803/su2697Isup3.cml

CCDC reference:

Additional supporting information:  crystallographic information; 3D view; checkCIF report

## Figures and Tables

**Table 1:** Hydrogen-bond geometry (Å, °)

*D*—H⋯*A*	*D*—H	H⋯*A*	*D*⋯*A*	*D*—H⋯*A*
N2—H2*A*⋯O3^i^	0.89 (4)	2.11 (4)	2.963 (3)	162 (3)
N2—H2*B*⋯O4^ii^	0.88 (3)	2.36 (3)	3.105 (3)	142 (3)
C15—H15⋯O1^iii^	0.95	2.38	3.307 (3)	166

Symmetry codes: (i) 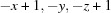; (ii) 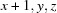; (iii) 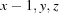.

**Fig. 1.**
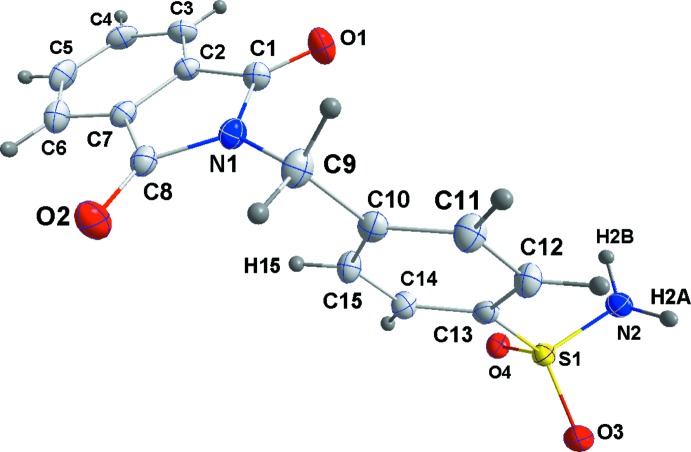
A view of the molecular structure of the title molecule, with atom labelling. Displacement ellipsoids are drawn at the 50% probability level.

**Fig. 2.**
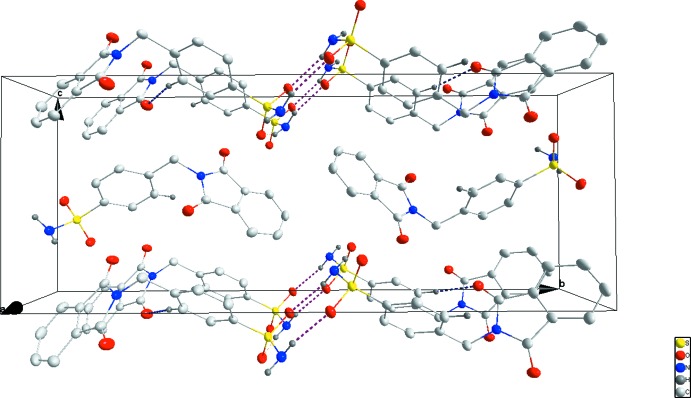
A view along a axis of the crystal packing of the title compound. The hydrogen bonds are shown as dashed lines (N—H···O purple; C—H···O green; see Table 1 for details).

## supplementary crystallographic information

### 1. Comment

Cyclic imides are an interesting class of compounds possessing important biological functions including anti-hyperlipidemic, anti-diabetic, anti-tumor and anti-inflammatory activities (Abdel-Aziz *et al.*, 2011*a*,*b*; Abdel-Aziz, 2007). As part of our ongoing studies in drug design and discovery, we report herein on the crystal structure of the title compound.

The title molecule, Fig. 1, is V-shaped with the mean plane of the isoindoline ring system (N1/C1-C8; r.m.s. deviation 0.006 Å) being inclined to the benzene ring (C10-C15) by 84.27 (13)°. The entire isoindoline-1,3-dione moiety is planar with the exception of atoms O1 and O2 which lie 0.013 (2) and 0.030 (2) Å, respectively, out of the mean plane of the carbon and nitrogen atoms.

In the crystal, inversion dimers are formed via N2—H2A···O3 hydrogen bonds (Table 1). These units associate further into corrugated ribbons *via* pairwise N2—H2B···O4 and C15—H15···O1 hydrogen bonds (Table 1 and Fig. 2). The ribbons run in the *a* direction and are parallel to (001).

### 2. Experimental

The synthesis of the title compound has been reported previously (Abdel-Aziz *et al.*, 2011*a*). A solution of 4-(aminomethyl)benzene-1-sulfonamide (10 mmol) and phthalic anhydride (10 mmol) in glacial acetic acid (10 ml) was heated under reflux for 6 h. After evaporation of the reaction mixture to dryness under reduced pressure, the residue was neutralized using aqueous sodium bicarbonate solution (4%) until effervescence ceased. The precipitate obtained was washed with water, dried *in vacuo* and recrystallized from methanol yielding colourless plate-like crystals.

### 3. Refinement

The NH_2_ H atoms were located in a difference electron-density map and freely refined. The C bound H atoms were included in calculated positions and treated as riding atoms: C—H = 0.95 and 0.99 Å for CH and CH_2_H atoms, respectively, with *U*_iso_(H) = 1.2*U*_eq_(C).

### Crystal data

**Table:**

C_15_H_12_N_2_O_4_S	*F*(000) = 656
*M**_r_* = 316.33	*D*_x_ = 1.548 Mg m^−^^3^
Monoclinic, *P*2_1_/*n*	Cu *K*α radiation, λ = 1.54178 Å
*a* = 4.9803 (1) Å	Cell parameters from 9982 reflections
*b* = 26.5291 (7) Å	θ = 3.3–69.7°
*c* = 10.2740 (3) Å	µ = 2.33 mm^−^^1^
β = 90.804 (1)°	*T* = 100 K
*V* = 1357.30 (6) Å^3^	Plate, colourless
*Z* = 4	0.27 × 0.07 × 0.02 mm

### Data collection

**Table:**

Bruker D8 VENTURE PHOTON 100 CMOS diffractometer	2533 independent reflections
Radiation source: INCOATEC IµS micro–focus source	2277 reflections with *I* > 2σ(*I*)
Mirror monochromator	*R*_int_ = 0.036
Detector resolution: 10.4167 pixels mm^-1^	θ_max_ = 69.7°, θ_min_ = 3.3°
ω scans	*h* = −6→5
Absorption correction: multi-scan (*SADABS*; Bruker, 2012)	*k* = −32→32
*T*_min_ = 0.85, *T*_max_ = 0.95	*l* = −12→12
22413 measured reflections	

### Refinement

**Table:**

Refinement on *F*^2^	Primary atom site location: structure-invariant direct methods
Least-squares matrix: full	Secondary atom site location: difference Fourier map
*R*[*F*^2^ > 2σ(*F*^2^)] = 0.042	Hydrogen site location: mixed
*wR*(*F*^2^) = 0.097	H atoms treated by a mixture of independent and constrained refinement
*S* = 1.17	*w* = 1/[σ^2^(*F*_o_^2^) + (0.007*P*)^2^ + 2.7892*P*] where *P* = (*F*_o_^2^ + 2*F*_c_^2^)/3
2533 reflections	(Δ/σ)_max_ < 0.001
207 parameters	Δρ_max_ = 0.35 e Å^−^^3^
60 restraints	Δρ_min_ = −0.48 e Å^−^^3^

### Special details

**Table:**

Geometry. All e.s.d.'s (except the e.s.d. in the dihedral angle between two l.s. planes) are estimated using the full covariance matrix. The cell e.s.d.'s are taken into account individually in the estimation of e.s.d.'s in distances, angles and torsion angles; correlations between e.s.d.'s in cell parameters are only used when they are defined by crystal symmetry. An approximate (isotropic) treatment of cell e.s.d.'s is used for estimating e.s.d.'s involving l.s. planes.
Refinement. Refinement of *F*^2^ against ALL reflections. The weighted *R*-factor *wR* and goodness of fit *S* are based on *F*^2^, conventional *R*-factors *R* are based on *F*, with *F* set to zero for negative *F*^2^. The threshold expression of *F*^2^ > σ(*F*^2^) is used only for calculating *R*-factors(gt) *etc*. and is not relevant to the choice of reflections for refinement. *R*-factors based on *F*^2^ are statistically about twice as large as those based on *F*, and *R*- factors based on ALL data will be even larger. H-atoms attached to carbon were placed in calculated positions (C—H = 0.95 Å) and included as riding contributions with isotropic displacement parameters 1.2 times those of the attached atoms.

### Fractional atomic coordinates and isotropic or equivalent isotropic displacement parameters (Å^2^)

**Table:**

	*x*	*y*	*z*	*U*_iso_*/*U*_eq_	
S1	0.34446 (12)	0.06815 (2)	0.37785 (6)	0.01772 (16)	
O1	0.8787 (4)	0.29938 (7)	0.42647 (19)	0.0289 (5)	
O2	0.2038 (4)	0.33953 (7)	0.70508 (18)	0.0270 (4)	
O3	0.2231 (4)	0.03320 (7)	0.46699 (18)	0.0234 (4)	
O4	0.1931 (4)	0.08113 (7)	0.26280 (17)	0.0228 (4)	
N1	0.5522 (4)	0.30797 (8)	0.5821 (2)	0.0196 (5)	
N2	0.6245 (5)	0.04320 (9)	0.3351 (2)	0.0213 (5)	
H2A	0.699 (7)	0.0253 (13)	0.399 (3)	0.036 (9)*	
H2B	0.734 (7)	0.0629 (13)	0.291 (3)	0.035 (9)*	
C1	0.6940 (5)	0.32243 (10)	0.4720 (2)	0.0203 (5)	
C2	0.5700 (5)	0.37076 (10)	0.4278 (2)	0.0195 (5)	
C3	0.6315 (5)	0.40119 (10)	0.3228 (3)	0.0250 (6)	
H3	0.7724	0.3928	0.2653	0.030*	
C4	0.4774 (6)	0.44466 (10)	0.3052 (3)	0.0277 (6)	
H4	0.5141	0.4665	0.2344	0.033*	
C5	0.2713 (6)	0.45654 (10)	0.3897 (3)	0.0291 (6)	
H5	0.1695	0.4864	0.3754	0.035*	
C6	0.2107 (6)	0.42563 (10)	0.4950 (3)	0.0254 (6)	
H6	0.0700	0.4337	0.5528	0.030*	
C7	0.3644 (5)	0.38266 (9)	0.5114 (2)	0.0194 (5)	
C8	0.3516 (5)	0.34265 (10)	0.6129 (2)	0.0195 (5)	
C9	0.6122 (6)	0.26288 (10)	0.6576 (3)	0.0224 (6)	
H9A	0.5115	0.2643	0.7400	0.027*	
H9B	0.8060	0.2627	0.6803	0.027*	
C10	0.5424 (5)	0.21422 (10)	0.5873 (2)	0.0200 (5)	
C11	0.6909 (5)	0.17103 (10)	0.6137 (3)	0.0229 (6)	
H11	0.8343	0.1723	0.6757	0.027*	
C12	0.6324 (5)	0.12619 (10)	0.5508 (3)	0.0222 (6)	
H12	0.7363	0.0969	0.5682	0.027*	
C13	0.4197 (5)	0.12451 (9)	0.4619 (2)	0.0179 (5)	
C14	0.2657 (5)	0.16688 (10)	0.4370 (3)	0.0212 (6)	
H14	0.1191	0.1653	0.3769	0.025*	
C15	0.3270 (5)	0.21161 (10)	0.5003 (3)	0.0229 (6)	
H15	0.2208	0.2407	0.4841	0.027*	

### Atomic displacement parameters (Å^2^)

**Table:**

	*U*^11^	*U*^22^	*U*^33^	*U*^12^	*U*^13^	*U*^23^
S1	0.0184 (3)	0.0157 (3)	0.0191 (3)	−0.0011 (2)	−0.0001 (2)	0.0007 (2)
O1	0.0278 (11)	0.0321 (11)	0.0269 (10)	0.0094 (9)	0.0047 (8)	0.0021 (9)
O2	0.0296 (10)	0.0318 (11)	0.0197 (10)	0.0032 (8)	0.0056 (8)	0.0010 (8)
O3	0.0224 (10)	0.0206 (9)	0.0272 (10)	−0.0019 (7)	0.0032 (8)	0.0046 (8)
O4	0.0249 (10)	0.0214 (9)	0.0219 (9)	−0.0009 (7)	−0.0054 (7)	0.0002 (8)
N1	0.0235 (11)	0.0175 (11)	0.0177 (11)	0.0008 (9)	−0.0002 (9)	0.0004 (9)
N2	0.0221 (12)	0.0201 (12)	0.0218 (12)	−0.0002 (9)	0.0009 (10)	0.0003 (10)
C1	0.0221 (13)	0.0216 (13)	0.0171 (13)	0.0005 (10)	−0.0007 (10)	−0.0004 (10)
C2	0.0197 (13)	0.0201 (13)	0.0185 (13)	−0.0024 (10)	−0.0027 (10)	−0.0014 (10)
C3	0.0240 (14)	0.0282 (15)	0.0228 (14)	−0.0051 (11)	−0.0005 (11)	0.0029 (12)
C4	0.0346 (16)	0.0235 (14)	0.0249 (14)	−0.0083 (12)	−0.0063 (12)	0.0068 (12)
C5	0.0393 (17)	0.0173 (13)	0.0303 (15)	0.0034 (12)	−0.0098 (13)	0.0008 (12)
C6	0.0302 (15)	0.0219 (14)	0.0239 (14)	0.0061 (11)	−0.0043 (11)	−0.0042 (11)
C7	0.0223 (13)	0.0176 (12)	0.0183 (13)	−0.0009 (10)	−0.0044 (10)	−0.0008 (10)
C8	0.0220 (13)	0.0185 (13)	0.0178 (13)	−0.0001 (10)	−0.0026 (10)	−0.0043 (10)
C9	0.0288 (14)	0.0195 (13)	0.0189 (13)	0.0029 (11)	−0.0031 (11)	0.0021 (10)
C10	0.0243 (13)	0.0204 (13)	0.0155 (12)	0.0008 (10)	0.0022 (10)	0.0006 (10)
C11	0.0269 (14)	0.0226 (13)	0.0189 (13)	0.0022 (11)	−0.0058 (11)	0.0024 (11)
C12	0.0247 (14)	0.0186 (13)	0.0232 (14)	0.0037 (11)	−0.0035 (11)	0.0018 (11)
C13	0.0196 (13)	0.0176 (12)	0.0165 (12)	−0.0015 (10)	0.0029 (10)	0.0023 (10)
C14	0.0213 (13)	0.0223 (13)	0.0199 (13)	0.0006 (10)	−0.0027 (10)	0.0016 (11)
C15	0.0258 (14)	0.0192 (13)	0.0237 (14)	0.0044 (11)	−0.0028 (11)	0.0005 (11)

### Geometric parameters (Å, º)

**Table:**

S1—O4	1.4350 (18)	C5—C6	1.394 (4)
S1—O3	1.4419 (18)	C5—H5	0.9500
S1—N2	1.610 (2)	C6—C7	1.382 (4)
S1—C13	1.764 (3)	C6—H6	0.9500
O1—C1	1.205 (3)	C7—C8	1.490 (4)
O2—C8	1.211 (3)	C9—C10	1.517 (4)
N1—C1	1.396 (3)	C9—H9A	0.9900
N1—C8	1.397 (3)	C9—H9B	0.9900
N1—C9	1.454 (3)	C10—C15	1.388 (4)
N2—H2A	0.89 (4)	C10—C11	1.389 (4)
N2—H2B	0.88 (3)	C11—C12	1.383 (4)
C1—C2	1.491 (4)	C11—H11	0.9500
C2—C7	1.382 (4)	C12—C13	1.390 (4)
C2—C3	1.385 (4)	C12—H12	0.9500
C3—C4	1.396 (4)	C13—C14	1.382 (4)
C3—H3	0.9500	C14—C15	1.386 (4)
C4—C5	1.390 (4)	C14—H14	0.9500
C4—H4	0.9500	C15—H15	0.9500
			
O4—S1—O3	117.22 (11)	C6—C7—C2	121.7 (2)
O4—S1—N2	108.72 (12)	C6—C7—C8	130.1 (2)
O3—S1—N2	106.35 (12)	C2—C7—C8	108.1 (2)
O4—S1—C13	107.77 (11)	O2—C8—N1	125.2 (2)
O3—S1—C13	108.78 (11)	O2—C8—C7	128.9 (2)
N2—S1—C13	107.65 (12)	N1—C8—C7	105.9 (2)
C1—N1—C8	111.9 (2)	N1—C9—C10	113.7 (2)
C1—N1—C9	123.8 (2)	N1—C9—H9A	108.8
C8—N1—C9	124.3 (2)	C10—C9—H9A	108.8
S1—N2—H2A	112 (2)	N1—C9—H9B	108.8
S1—N2—H2B	116 (2)	C10—C9—H9B	108.8
H2A—N2—H2B	116 (3)	H9A—C9—H9B	107.7
O1—C1—N1	125.0 (2)	C15—C10—C11	119.3 (2)
O1—C1—C2	129.3 (2)	C15—C10—C9	121.2 (2)
N1—C1—C2	105.7 (2)	C11—C10—C9	119.4 (2)
C7—C2—C3	121.7 (2)	C12—C11—C10	120.7 (2)
C7—C2—C1	108.3 (2)	C12—C11—H11	119.7
C3—C2—C1	130.0 (2)	C10—C11—H11	119.7
C2—C3—C4	117.1 (3)	C11—C12—C13	119.3 (2)
C2—C3—H3	121.5	C11—C12—H12	120.4
C4—C3—H3	121.5	C13—C12—H12	120.4
C5—C4—C3	121.1 (3)	C14—C13—C12	120.8 (2)
C5—C4—H4	119.5	C14—C13—S1	119.0 (2)
C3—C4—H4	119.5	C12—C13—S1	120.22 (19)
C4—C5—C6	121.4 (3)	C13—C14—C15	119.4 (2)
C4—C5—H5	119.3	C13—C14—H14	120.3
C6—C5—H5	119.3	C15—C14—H14	120.3
C7—C6—C5	117.0 (3)	C14—C15—C10	120.6 (2)
C7—C6—H6	121.5	C14—C15—H15	119.7
C5—C6—H6	121.5	C10—C15—H15	119.7
			
C8—N1—C1—O1	179.0 (3)	C2—C7—C8—O2	178.4 (3)
C9—N1—C1—O1	0.7 (4)	C6—C7—C8—N1	179.6 (3)
C8—N1—C1—C2	−0.5 (3)	C2—C7—C8—N1	−0.9 (3)
C9—N1—C1—C2	−178.9 (2)	C1—N1—C9—C10	−70.0 (3)
O1—C1—C2—C7	−179.6 (3)	C8—N1—C9—C10	111.9 (3)
N1—C1—C2—C7	−0.1 (3)	N1—C9—C10—C15	−32.3 (4)
O1—C1—C2—C3	1.0 (5)	N1—C9—C10—C11	149.9 (2)
N1—C1—C2—C3	−179.5 (3)	C15—C10—C11—C12	2.4 (4)
C7—C2—C3—C4	0.3 (4)	C9—C10—C11—C12	−179.8 (2)
C1—C2—C3—C4	179.7 (3)	C10—C11—C12—C13	−1.0 (4)
C2—C3—C4—C5	−0.1 (4)	C11—C12—C13—C14	−0.6 (4)
C3—C4—C5—C6	0.1 (4)	C11—C12—C13—S1	178.8 (2)
C4—C5—C6—C7	−0.1 (4)	O4—S1—C13—C14	20.6 (2)
C5—C6—C7—C2	0.2 (4)	O3—S1—C13—C14	−107.4 (2)
C5—C6—C7—C8	179.7 (3)	N2—S1—C13—C14	137.7 (2)
C3—C2—C7—C6	−0.3 (4)	O4—S1—C13—C12	−158.8 (2)
C1—C2—C7—C6	−179.8 (2)	O3—S1—C13—C12	73.2 (2)
C3—C2—C7—C8	−179.9 (2)	N2—S1—C13—C12	−41.7 (2)
C1—C2—C7—C8	0.6 (3)	C12—C13—C14—C15	0.8 (4)
C1—N1—C8—O2	−178.4 (2)	S1—C13—C14—C15	−178.5 (2)
C9—N1—C8—O2	−0.1 (4)	C13—C14—C15—C10	0.5 (4)
C1—N1—C8—C7	0.9 (3)	C11—C10—C15—C14	−2.1 (4)
C9—N1—C8—C7	179.2 (2)	C9—C10—C15—C14	−179.9 (2)
C6—C7—C8—O2	−1.2 (5)		

### Hydrogen-bond geometry (Å, º)

**Table:**

*D*—H···*A*	*D*—H	H···*A*	*D*···*A*	*D*—H···*A*
N2—H2*A*···O3^i^	0.89 (4)	2.11 (4)	2.963 (3)	162 (3)
N2—H2*B*···O4^ii^	0.88 (3)	2.36 (3)	3.105 (3)	142 (3)
C15—H15···O1^iii^	0.95	2.38	3.307 (3)	166

Symmetry codes: (i) −*x*+1, −*y*, −*z*+1; (ii) *x*+1, *y*, *z*; (iii) *x*−1, *y*, *z*.
